# The effect of keratinized mucosa on the severity of peri-implant mucositis differs between periodontally healthy subjects and the general population: a cross-sectional study

**DOI:** 10.1007/s00784-020-03422-1

**Published:** 2020-07-01

**Authors:** Laila Kabir, Meike Stiesch, Jasmin Grischke

**Affiliations:** Department of Prosthetic Dentistry and Biomedical Materials Research, Hannover Medical School, Hannover, Germany

**Keywords:** Peri-implantitis, Mucositis, Periodontitis, Dental implant, Observational study

## Abstract

**Objective:**

The study aims to investigate the effect of reduced keratinized mucosa (KM) and other risk indicators on the severity of peri-implant mucositis in (i) the general population, (ii) in periodontally healthy patients, and (iii) in periodontally healthy patients without a history of periodontitis.

**Materials and methods:**

Anamnesis and the following clinical parameters were taken: mucosal-index, bleeding on probing, local plaque index, oral hygiene-index, and width of KM. Mucositis severity score was determined for each implant. Multi-level and subgroup analysis was performed on the patient and implant level.

**Results:**

Six hundred twelve implants in 130 patients were analyzed. Subgroup analysis showed significant associations between KM < 2 mm and the severity score in (ii) periodontally healthy patients (*p* = 0.014) and in (iii) patients without history of periodontitis (*p* = 0.017). Secondary outcome showed higher severity scores for patients with insufficient oral hygiene or without residual teeth (*p* ≤ 0.001), in maxillary implants (*p* = 0.04), and for the number of implants per patient (*p* ≤ 0.001).

**Conclusion:**

Within the limits of the study, one may conclude that a reduced width of KM is a risk indicator for the severity of peri-implant mucositis in periodontally healthy patients and patients without a history of periodontitis.

**Clinical relevance:**

The results indicate a band of ≥ 2 mm KM to reduce the severity of peri-implant mucositis in periodontally healthy patients.

## Introduction

Dental implants have become a widespread implemented therapy to replace missing teeth or to install fixed or partially removable dentures, solving a number of esthetic and functional problems in contemporary prosthetics and achieving high satisfaction rates among patients [1–3]. However, biological complications of the implant surrounding tissue, such as peri-implantitis and peri-implant mucositis, are common [3–5].

Dysbiosis of the oral microbiome seems to be the etiologic factor initiating peri-implant disease and leading to the inflammatory response resulting in pocket formation due to loss of peri-implant bone and leading ultimately to implant loss [6, 7]. Depending on the different disease definitions, the reported prevalence rates of peri-implantitis and peri-implant mucositis differ among studies. A recent meta-analysis found a prevalence of 50% for peri-implant mucositis and 15% for peri-implantitis [8].

Several risk indicators, such as smoking [1, 9, 10], presence of periodontitis or history of periodontitis [5, 9, 11–13] lack of oral hygiene [1, 11], maxillary implants [5, 14, 15] or location at a posterior region in the jaw, male gender [4, 5], an increased number of implants [16], and loading time or patient age [9], have been reported in the literature. Moreover, a protective effect of bone or soft tissue augmentation was reported [11]. Furthermore, an investigation with a 10-year observation time showed statistically higher mean marginal bone loss rates in periodontally compromised patients treated for periodontitis than periodontally healthy patients [12, 13]. The faciolingual dimension of the masticatory mucosa at the implant site, depending on genetic factors and crestal bone resorption, is decisive for the width of KM facing the implant surface [17]. The negative influence of a narrow width of KM on oral hygiene behavior due to higher brushing discomfort is often reported in the literature [18–20]. There is a general consensus that a reduced band of KM is associated with mucosal recession and consequently makes KM indispensable for satisfying esthetic results [14, 21–25]. Though the influence of KM on peri-implant health has been controversially discussed in the literature, the evidence remains equivocal [6]. Several studies have found a statistically significant association between a reduced width of KM and clinical parameters of soft tissue inflammation [4, 9, 21, 23, 26–32]. However, other studies found no association regarding the effect of KM on peri-implant health [9, 14, 33, 34].

Likewise, a meta-analysis found heterogenic results in the current literature on the association between reduced keratinized tissues and peri-implantitis and stated that further research is necessary to elucidate this research question [8].

Interestingly, Grischke et al. found a significant relation between a reduced width of KM and the severity of peri-implant mucositis applying a severity score in low-risk patients without history or presence of periodontitis [35]. Unfortunately, the study did not investigate the effect in the general population.

Consequently, the purpose of this study was to determine the influence of KM on the severity of peri-implant mucositis in (i) all patients, referred to as the general population including periodontally healthy patients and patients with (history of) periodontitis and in two subgroups of patients without (ii) presence and (iii) history of periodontitis. Moreover, as a secondary outcome, other implant- and patient-related putative risk indicators for severity of peri-implant mucositis are aimed to be identified in the different groups.

## Materials and methods

### Study design

This investigation is a mono-centric observational cross-sectional study conducted in 2019. The study from Grischke et al. [35] served as a basis for planning and sample size calculation. All procedures were approved by the ethics committee of Hannover Medical School (no. 8207_BO_S_2018).

In order to ensure the best practice quality of research, the current study adheres to the STROBE guidelines [36] for cross-sectional studies.

### Patient sample/study population

The sample size calculation was performed based on the results of the pilot study [35]. To test our hypothesis that supports a significant relation between insufficient KM and increased severity of peri-implant mucositis in periodontally healthy patients, we chose an alpha error rate of 5%. To reach a power of 80% with an estimated effect size of 0.324, a sample size *N* = 36 study participants for each group was calculated.

**Fig. 1:**
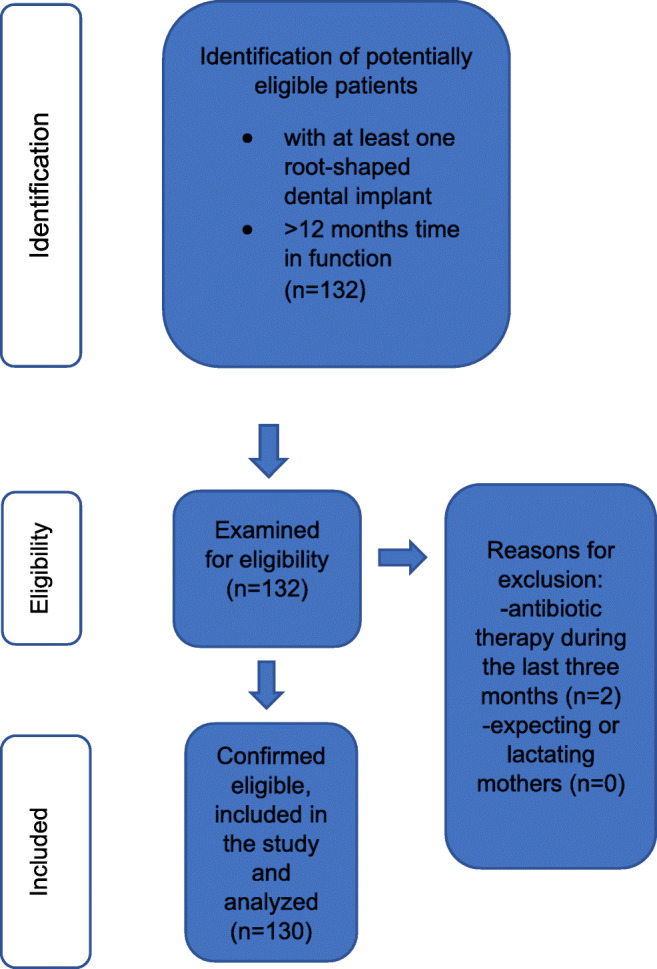
Flowchart of the patient inclusion and exclusion process. 130 patients were found eligible. Two patients were excluded during the 3-month recruitment process due to antibiotic therapy

All patients attending maintenance care at the Department of Prosthetic Dentistry and Biomedical Materials Science of Hannover Medical School with at least one root-shaped dental implant with more than 1 year of follow-up after loading were asked to participate in the study between February and May 2019. Inclusion criteria were at least one root-shaped dental implant with at least 1 year of follow-up. The exclusion criteria were patients with a history of antibiotic therapy during the last 3 months and expecting or lactating mothers. No further exclusion criteria were defined. Two study participants could not be included in the study because of recent antibiotic therapy (Fig. 1). All study participants were verbally informed prior to study registration. Their oral and written informed consent was given before study participation. The exclusion criteria of the first study are described in detail by the authors Grischke et al. [35]. Patient recruitment process and clinical assessments were performed by one calibrated examiner following a standardized protocol implying that all examinations were carried out with a maximum of consistency in regard to the case history, the order of examination procedures, probing pressure, and visual assessment.

### Clinical examination/clinical parameters

Preceding the clinical examination, detailed information was collected regarding the implant’s observation time, position, loading time, and previous bone augmentation. Further compromised immune system, alcohol or drug abuse, hyperglycemia, oral and maxillofacial tumor with history of head and neck radiation, smoking status, and allergies especially iodine hypersensitivity were enquired. Additionally, a history of periodontitis as well as age and gender was assessed.

Afterwards, the following clinical measurements were taken:

Probing depth (PD) was measured in millimeters at six implant sites (mesiobuccal, buccal, distobuccal, mesiolingual, lingual, distolingual) using a graduated periodontal probe (GY12, DEPPELER SA, Rolle, Switzerland) with an intended force of 0.2 N.

Bleeding on probing or suppuration (BoP/Sup) occurring within 30 s after measurement of PD was recorded.

The local plaque was evaluated by using the modified plaque index (mPl) [37] while the general hygiene behavior was assessed using a full-mouth plaque score approximal space plaque index (API, approximal space plaque index) expressed in percent [38]. In this process, all approximal spaces showing plaque were divided by the number of approximal spaces in both jaws.

For a deepened evaluation of the condition of peri-implant soft-tissues and conceivably inflammation, the mucositis-index (mGI) [39] was used.

The width of peri-implant KM was measured as the distance between the gingival margin and the muco-gingival junction. To improve visibility of the border between the keratinized and non-keratinized mucosa, iodine solution (Lugolsche Lösung 5%, vitalundfit GmBH, Jüchen, Germany) was applied to the mucosa if necessary [40].

For assessing the presence of periodontitis, the Community Periodontal Index (CPI), introduced by the American Academy of Periodontology and the American Dental association in 1992, was applied [41].

Data were collected following a standardized operating procedure and saved electronically.

### Definition of outcomes

KM was categorized into either sufficient or insufficient in order to attain analogous and comparable outcomes to the study conducted by Grischke et al. [35]. The dichotomous division of KM was conducted as described before. Briefly, a threshold value of < 2 mm of KM was considered as insufficient and KM ≥ 2 mm as sufficient [35].

In the statistical analysis, we distinguished three different groups: the general study population (i) including periodontally healthy patients or patients with presence and/or history of periodontitis; the first subgroup (ii) including periodontally healthy patients; and the second subgroup (iii) including periodontally healthy patients without a history of periodontitis.

Complying with the references of the Seventh European Consensus Workshop on Periodontology and the statements of the workgroup 4 of the 2017 World Workshop, the key parameter for peri-implant mucositis assumption is the presence of bleeding on gentle probing with or without concomitant suppuration and deepening of peri-implant pockets compared to previous examinations but necessarily with the absence of bone loss beyond crestal bone level changes resulting from initial bone remodeling. However peri-implantitis is defined by changes in the crestal bone level in conjunction with BoP and pus as a common finding in peri-implantitis sites [6, 42].

The general oral hygiene behavior assessed by the API was either categorized as adequate or inadequate with a cutoff value of 35%. An API ≥ 35% was considered inadequate oral hygiene [38]. Non-smokers were defined as never smokers and smokers who quit more than 5 years ago [43].

The variable mGI-BoP was adopted from the study from Grischke et al. [35] for the assessment of the severity of peri-implant mucositis.

The severity score consists of the mGI and BoP being assessed at six sites per implant each. Every site can get up to four points: a maximum of three points for the mGI plus one point when the site shows bleeding on gentle probing. As the severity score is measured at six sites per implant, the variable consequently reaches from 0 to 24 for each implant.

### Statistical analysis

First, a descriptive analysis of potential confounders was performed on patient level by recording frequencies, the mean and standard deviation. For patient-level analysis, only one implant, the worst implant, per patient was considered. The worst implant was assessed by the highest severity score of all implants of a patient. In the first step, the possible confounders were included in a univariate regression model to assess possible relations with the severity score. This was conducted on the patient and implant level.

The primary statistical analysis was again performed on the patient and implant level using an adjusted multivariate linear regression model analyzing the previously identified confounding variables and their relation to the dependent variable (mucositis severity score). *P* values ≤ 0.05 were considered statistically significant.

First, the general population (i) including all patients was analyzed in a multivariate regression model. Second, subgroup analysis was performed (ii) including only periodontally healthy patients and (iii) including only periodontally healthy patients without a history of periodontitis.

## Results

### Descriptive analysis

A total of 612 root-shaped dental implants in 130 patients were evaluated and examined for signs of peri-implant mucositis and the width of KM during a 4-month study period. The implants investigated were either from Straumann® Dental Implant System (Straumann GmbH, Freiburg, Germany) or Astra Tech Implant System (Dentsply Sirona Deutschland GmbH, Bensheim, Germany). The implant brand showed no significant effect on peri-implant health. The descriptive analysis is represented in Tables 1 and 2 and revealed that in 348 out of 612 implants, the width of KM was < 2 mm. Considering only the worst implant of every patient, 74 out of 130 participants thus 57% of the patient sample shows a lack of KM in the peri-implant tissue. The dataset comprises 71 female and 59 male participants with a mean age of 70 ± 10.32 years with a range between 38 and 92 years. Seventy-one percent of the study population had a history of periodontitis and 55% had, according to the CPI, at the time of examination periodontitis. A lack of general oral hygiene behavior was found in 63% of the patients, whereas only 8% were current smokers. No subjects with compromised immune system, alcohol or drug abuse, hyperglycemia, oral and maxillofacial tumor, or with history of head and neck radiation were identified.

**Table 1:** Descriptive analysis of patient characteristics on patient level

		Total (worst implant) *n* = 130	KM < 2 mm *n* = 74	KM ≥ 2 mm *n* = 56
Patient characteristics				
Mean age	Years (sd)	69.85 ± 10.32	70.03 ± 11.03	69.61 ± 9.39
Sex	Female	71	39 (54.9%)	32 (45.1%)
	Male	59	35 (59.3%)	24 (40.7%)
History of periodontitis	Yes	92	51 (55.4%)	41 (44.6%)
	No	38	23 (60.5%)	15 (39.5%)
Smoker	Yes	11	4 (36.4%)	7 (63.6%)
	No	119	70 (58.8%)	49 (41.2%)
Periodontitis	Yes	71	36 (50.7%)	35 (49.3%)
	No	59	38 (64.4%)	21 (35.6%)
API	> 35%	82	48 (58.5%)	34 (41.5%)
	< 35%	48	26 (54.2%)	22 (45.8%)
Residual teeth	Yes	116	65 (56%)	51 (44%)
	No	14	9 (64.3%)	5 (35.7%)
Number of implants	Mean (sd)	4.87 ± 3.01	7.72 ± 2.73	5.07 ± 3.38

**Table 2:** Descriptive analysis of the implant characteristics on patient level

		Total (worst implant) *n* = 130	KM < 2 mm *n* = 74	KM ≥ 2 mm *n* = 56
Implant characteristics				
Loading time	Year (sd)	10.15 ± 6.31	9.81 ± 6.55	10.58 ± 6.01
Region	Anterior	38	20 (52.6%)	18 (47.4%)
	Posterior	92	54 (58.7%)	38 (41.3%)
Bone augmentation	Yes	65	38 (58.5%)	27 (41.5%)
	No	65	36 (55.4%)	29 (44.6%)
Probing depth	mm (sd)	3.81 ± 1.72	3.92 ± 1.93	3.65 ± 1.38
Mean mPI	Grade (sd)	0.84 ± 0.86	0.86 ± 0.94	0.82 ± 0.75
Jaw	OK	69	29 (42%)	40 (58%)
	UK	61	45 (73.8%)	16 (26.2%)
Mean mBI	Grade (sd)	1.42 ± 0.88	1.42 ± 0.89	1.41 ± 0.87
BoP	0–6 (sd)	3.48 ± 2.05	3.35 ± 2.02	3.64 ± 2.1
Mean mGI	Grade (sd)	1.49 ± 0.73	1.52 ± 0.7	1.46 ± 0.76
BoP	Yes	119	70 (58.8%)	49 (41.2%)
	No	11	4 (36.4%)	7 (63.6%)

The implants were in function for an average of 10.15 ±6.31 years with a range between 1 and 31 years. The mean PD on the patient level is 3.81 mm. Seventy-one percent of 612 implants are located in the posterior region including the premolar and molar region. Only eleven patients have a healthy peri-implant mucosa showing no presence of BoP at any site.

**Fig. 2:**
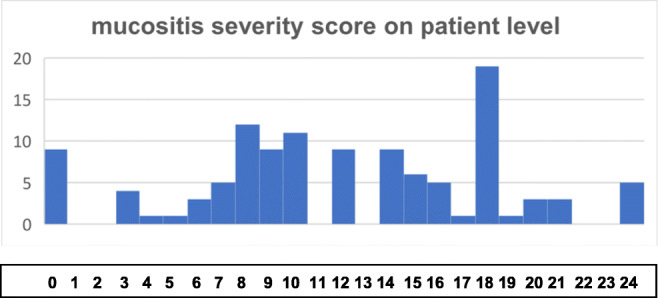
The mucositis severity score is a sum of the mucositis score (mGI from 0 to 3) and the BoP (0/1) and reaches up to 4 points at each implant site. The severity score is calculated at six implant sites and reaches from 0 (no visual signs of inflammation and no bleeding on probing at any implant site) up to 24 points (severe inflammation with spontaneous bleeding/ulceration as well as BoP at six implant sites). The scores from 0 to 24 are represented in the horizontal axis. The vertical axis represents the absolute amount of implants in the present cohort

The distribution of the scores on the patient level is shown in Fig. 2. A score of 18 was found to be the most frequent severity score among all included implants.

### Univariate and multivariate regression on patient and implant level

Univariate regression analysis revealed that all possible risk indicators were associated with our primary outcome the severity score, so was the exposition of interest insufficient KM (patient level, 95% CI (9.2–15.2), *p* ≤ 0.001; implant level, 95% CI (7–9.2), *p* ≤ 0.001). Consequently, all variables were used in the adjusted sensitivity analysis of the primary objective. There was (i) no statistical significance between insufficient KM and the severity score in the general population on patient and implant level analysis, respectively (patient level, 95% CI (− 0.6–2.7), *p* = 0.195; implant level, 95% CI (− 0.8–0.9), *p* = 0.962). Multivariate analysis in the general population (i) revealed significantly higher severity scores with increasing PD (patient level, 95% CI (0.9–1.9), *p* ≤ 0.001; implant level, 95% CI (1.4–2), *p* ≤ 0.001) and in patients with no residual teeth (patient level, 95% CI (− 8.5–2.8), *p* ≤ 0.001; implant level, 95% CI (1.9 to − 1), *p* ≤ 0.001) or in insufficient oral hygiene (API > 35%; patient level, 95% CI (2.5–6.2), *p* ≤ 0.001; implant level, 95% CI (− 3.7–3.9), *p* ≤ 0.001) on patient and implant level. Maxillary implants (95% CI (0.1–3.4), *p* = 0.04) and the number of implants (95% CI (0.3–0.9), *p* ≤ 0.001) were associated with significantly higher severity scores on the patient level and male gender (95% CI (− 1.8 to − 0.3), *p* = 0.008) was a risk indicator for higher severity scores on implant level. All results of the univariate and multivariate analysis including all patients are depicted in Table 3. Subgroup analysis showed a significant association between insufficient KM and the severity score in subjects without (ii) presence or (iii) history of periodontitis on implant level and patient level (implant level, 95% CI (0.3–2.9), *p* = 0.017; patient level 95% CI (2.1–13.6), *p* = 0.014). All statistical outcomes of groups ii and iii are depicted in Table 4.

**Table 3:** Univariate and multivariate results for all putative risk indicators with the target variable mGI-BoP on patient level (PL) and implant level (IL) including all patients (*n* = 130). Significant *p* values are marked in italics

		Univariate analysis			Multivariate analysis		
Risk indicator		*n* = 130 patients/*n* = 612 implants			*n* = 130 patients/*n* = 612 implants		
		*t* statistics	CI (95%)	*p* value	*t* statistics	CI (95%)	*p* value
KM < 2 mm	PL	8.087	9.209–15.183	*< 0.001*	1.188	− 0.558–2.705	0.195
	IL	14.359	6.987–9.202	*< 0.001*	1.303	− 0.845–0.887	0.962
Loading time	PL	14.466	0.796–1.049	*< 0.001*	0.704	− 0.167–0.177	0.73
	IL	22.340	0.509–0.607	*< 0.001*	− 0.346	− 0.048–0.093	0.536
Region	PL	14.441	5.196–6.847	*< 0.001*	0.208	− 0.828–1.011	0.844
	IL	23.232	3.635–4.306	*< 0.001*	0.197	− 0.139–0.737	0.18
Maxilla	PL	183.104	6.713–8.342	*< 0.001*	1.683	0.076–3.371	*0.04*
	IL	29.914	4.921–5.613	*< 0.001*	2.072	− 0.407–1.236	0.322
Bone augmentation	PL	8.401	8.801–14.232	*< 0.001*	0.300	− 1.889–1.416	0.777
	IL	16.263	7.074–9.018	*< 0.001*	− 0.284	− 1.364–0.323	0.226
Probing depth	PL	24.128	2.759–3.252	*< 0.001*	4.772	0.898–1.853	*< 0.001*
	IL	38.519	2.261–2.504	*< 0.001*	5.706	1.435–2.015	*< 0.001*
mPI	PL	11.755	7.053–9.911	*< 0.001*	1.627	− 0.313–1.794	0.167
	IL	21.169	5.374–6.474	*< 0.001*	1.392	− 0.205–1.407	*0.001*
API > 35%	PL	23.012	6.439–7.647	*< 0.001*	3.263	2.473–6.181	*< 0.001*
	IL	36.143	4.679–5.217	*< 0.001*	4.623	− 3.704–3.861	*< 0.001*
Number of implants	PL	20.067	1.191–2.331	*< 0.001*	4.274	0.314–0.857	*< 0.001*
	IL	26.77	0.053–1.104	*< 0.001*	− 1.205	0.357–0.049	< 0.229
No residual teeth	PL	15.628	10.436–13.461	*< 0.001*	− 3.917	− 8.457–2.777	*< 0.001*
	IL	26.725	7.73–8.957	*< 0.001*	− 3.43	1.883 to − 1.007	*< 0.001*
Age	PL	21.478	0.154–0.185	*< 0.001*	− 0.594	− 0.069–0.053	0.805
	IL	33.844	0.11–0.124	*< 0.001*	0.247	− 0.02–0.052	0.375
Male gender	PL	15.744	5.985–7.707	*< 0.001*	− 1.656	1.59–1.287	0.835
	IL	26.150	4.476–5.203	*< 0.001*	− 2.09	− 1.796 to − 0.273	*0.008*
Smoker	PL	2.56	2.686–21.064	*< 0.012*	0.484	− 3.091–2.782	0.917
	IL	4.573	3.967–9.94	*< 0.001*	− 0.104	− 2.04–0.737	0.357
History of periodontitis	PL	13.070	10.675–14.489	*< 0.001*	− 1.382	− 3.2–0.57	0.17
	IL	24.802	8.021–9.401	*< 0.001*	− 1.101	− 1.627–2.458	0.272
Periodontitis	PL	12.956	11.203–15.247	*< 0.001*	1.402	− 0.528–3.087	0.164
	IL	23.846	8.150–9.614	*< 0.001*	− 0.85	− 1.412–0.559	0.396

**Table 4:** Multivariate results of the subgroup analysis for all putative risk indicators with the target variable mGI-BoP on patient level (PL) and implant level (IL) including periodontally healthy (*n* = 59) and patients without presence and history of periodontitis (*n* = 21). Significant *p* values are marked in italics

		Multivariate analysis without patients with periodontitis			Multivariate analysis without patients with (history of) periodontitis		
Risk indicator		*n* = 59/*n* = 264 implants			*n* = 21 patients/*n* = 79 implants		
		*t* statistics	CI (95%)	*p* value	*t* statistics	CI (95%)	*p* value
KM < 2 mm	PL	0.038	− 2.286–2.374	0.97	3.150	2.102–13.586	*0.014*
	IL	2.392	0.284–2.926	*0.017*	0.565	− 0.216–3.346	0.084
Loading time	PL	0.356	− 0.183–0.262	0.723	2.384	0.027–1.612	*0.044*
	IL	2.091	0.007–0.238	*0.038*	− 0.62	− 0.261–0.137	0.537
Region	PL	− 2.089	− 2.622 to − 0.048	*0.042*	− 0.360	− 3.58–2.613	0.728
	IL	0.356	− 0.537–0.774	0.722	− 0.738	− 1.706–0.785	0.463
Maxilla	PL	1.553	− 0.529–4.092	0.127	2.401	0.209–10.392	*0.043*
	IL	0.296	− 1.079–1.46	0.768	2.095	0.088–3.693	*0.04*
Bone augmentation	PL	− 0.736	− 3.412–1.585	0.465	4.545	7.532–23.049	*0.002*
	IL	− 1.179	− 2.145–0.539	0.24	1.27	− 0.991–4.452	0.209
Probing depth	PL	4.708	0.824–2.056	*< 0.001*	2.761	0.234–2.602	*0.025*
	IL	8.835	1.638–2.577	*< 0.001*	5.586	1.24–2.62	*< 0.001*
mPI	PL	1.033	− 0.770–2.393	0.307	− 0.918	− 5.495–2.365	0.385
	IL	2.534	0.249–1.986	*0.012*	− 0.852	− 2.441–0.981	0.398
API > 35%	PL	2.885	1.068–6.009	*0.006*	3.619	2.166–9.772	*0.007*
	IL	4.432	1.927–5.009	*< 0.001*	2.922	0.984–5.23	*0.005*
Number of implants	PL	4.616	0.516–1.314	*0.001*	− 2.035	− 2.072–0.13	0.076
	IL	0.721	− 0.127–0.273	0.471	0.919	− 0.23–0.623	0.361
No residual teeth	PL	− 2.721	− 6.921 to − 1.033	*0.009*	− 0.978	− 10.914–4.414	0.357
	IL	− 2.134	− 3.059 to − 0.122	*0.034*	1.055	− 1.792–5.809	0.296
Age	PL	− 0.696	− 0.109–0.053	0.49	− 2.273	− 0.387–0.003	0.053
	IL	− 1.26	− 0.082–0.018	0.209	− 1.561	− 0.169–0.021	0.123
Male gender	PL	0.549	− 1.495–2.614	0.586	− 2.06	− 8.513–0.48	0.073
	IL	− 2.534	− 2.82 to − 0.354	*0.012*	− 2.217	− 4.223 to − 0.221	*0.03*
Smoker	PL	− 1.509	− 6.255–0.897	0.138	− 4.668	− 20.536 to − 6.954	*0.002*
	IL	− 2.677	− 4.085–− 0.622	*0.008*	− 3.682	− 8.445 to − 2.507	*< 0.001*
History of periodontitis	PL	0.12	− 2.556–2.879	0.905			
	IL	− 0.533	− 2.077–1.193	0.595			

## Discussion

The purpose of this study was to determine the influence of KM on the severity of peri-implant mucositis in the general population (i), including periodontally healthy patients and patients with (history of) periodontitis and in two subgroups of patients without (ii) presence and without (iii) history of periodontitis.

Our hypothesis that insufficient width of KM is a risk indicator for the severity of peri-implant mucositis was accepted for the subgroup of patients without (ii) presence and without (iii) history of periodontitis since a statistically significant influence of KM was proven on the implant and patient level.

The authors believe that the knowledge of putative risk factors threatening peri-implant health is essential for the development of efficient algorithms for the prevention, diagnosis, and therapy of peri-implant diseases [44].

The width of KM was measured on the mid-facial and mid-lingual site. The sites were graduated into either sufficient (KM ≥ 2 mm) or insufficient (KM < 2 mm). This dichotomous division has been used numerous times in literature [23, 26]. In accordance with the former investigations conducted by Grischke et al. [35], the implants had to show a minimum of 2 mm KM at the buccal and the lingual site to be graduated with sufficient KM in the present study. In line with our current findings, Schrott et al. discovered a statistically significant association between a minimum of 2 mm of KM and reduced plaque accumulation and bleeding tendencies in patients exercising good oral hygiene [23]. The authors believe that a lack of KM simplifies plaque accumulation due to the increased mobility of lining mucosa facilitating plaque accumulation in the pockets. Moreover, plaque accumulation as a result of inadequate oral hygiene behavior around dental implants with reduced KM is to be related to higher brushing discomfort [18–20]. However, discomfort as an individual sensation is not objectively measurable and thus complicates scientific research on that topic. This could be a reason for studies reporting no brushing discomfort around dental implants showing reduced or no width of KM [28, 45].

The authors believe that a dichotomous evaluation of the clinical parameter BoP is not precise enough to account for the severity of inflammation [35] and should be accompanied by visual inspection of the peri-implant tissue [6]. Not only the presence but especially the degree of severity of the peri-implant mucositis may possibly be accompanied by an elevated risk to develop peri-implantitis in the future. This will be of interest in future research as the positive predictive value of BoP for disease progression from peri-implant mucositis to peri-implantitis has not been clarified to the knowledge of the authors until today.

The histogram depicted in Fig. 2 shows the different points of the severity score from 0 to 24 and the belonging absolute amount of implants on the patient level. The highest column and hence the most frequent severity score is score 18. It consists of a positive BoP on each site (6 points) and an mGI of 2 for each site showing no signs of ulceration but typical signs of inflammation like swelling or erythema. Thus, score 18 stands for a pronounced inflammation throughout the peri-implant mucosa with bleeding and visual signs of inflammation. This condition is marked by an absence of bone loss displaying a clear demarcation of the state of peri-implantitis. However, this outstanding column potentially marks the beginning of severe peri-implant mucositis with a positive predictive value for peri-implantitis.

Moreover, columns 8, 9, and 10 are high and striking. They represent half of the score 18 and stand hence for mucositis of 50% of the tissue or more precisely for the inflammation of a mesial or distal space. This degree of inflammation corresponds with moderate inflammation. The scores below show low signs of inflammation, with a maximum of two sites of positive BoP. Severity scores below 8 may be misjudged as peri-implant mucositis but may only show positive BoP due to the stimulus of probing because of their narrow and tight mucosa and not as a result of peri-implant inflammation.

Regarding former studies having carried out thematically comparable investigations, it is important to mention that, in most other studies, the presence and not the severity of peri-implant mucositis has been measured and analyzed.

Matarazzo et al. also investigated the severity of peri-implant diseases but defined only severe peri-implantitis by the extent of marginal bone loss and therefore focused fewer on the severity of peri-implant mucositis. However, also peri-implantitis is characterized by mucosal inflammation and therefore makes the results of Matarazzo et al. considerable at this point. In accordance with our results, they showed a significant association between the event peri-implantitis and male gender, maxillary implants, an increased number of implants and a width of KM < 2 mm [4].

Other studies verified a significant association between a reduced width of KM and peri-implant disease though the cutoff points determining the sufficiency of KM varies among the studies [28, 30, 32]. Compatible to our results, Ueno et al. found a statistical relation between a reduced width of KM (< 2 mm) and PD, BoP, and presence of plaque. Interestingly, the authors could not confirm the results mentioned above for a reduced width of keratinized gingiva (< 2 mm) around periodontal tissues giving evidence that the significant relation between a reduced width of KM and signs of peri-implant mucositis is restricted on implants and is not transferable to natural teeth [28].

However, different authors have investigated the effect of the presence of KM on peri-implant mucositis and found even contradictory events [9, 33]. Ross-Jansåker et al. stated that the presence of KM is statistically significantly associated with peri-implant mucositis. In this context, it is important to keep in mind that peri-implant mucositis was defined in their study as the presence of BoP and PD ≥ 4 mm. Considering the general consensus among many authors that a reduced band of KM is associated with mucosal recession and therefore prohibiting mucosal pocket formation, the different case definition in that study requiring an increased PD for the assessment of peri-implant mucositis may be explanatory for contradictory results.

Numerous studies have reported that the presence and history of periodontitis have a significant negative effect on peri-implant health. Boynuegri et al. investigated implants in edentulous patients and thus analyzed like this cross-sectional study patients without current periodontitis. They found out a significant relation between a reduced zone of KM (KM < 2 mm) and mucosal inflammation, plaque formation, and pro-inflammatory mediators [26]. It seems essential to eliminate periodontitis as confounding variable when aiming to analyze the effect of KM on peri-implant health as patients eligible for soft tissue augmentation are those low-risk patients with sufficient oral hygiene and without presence of periodontitis. The analysis of the subgroup in the current study excluding patients with presence of periodontitis confirmed the findings of the study conducted by Grischke et al. among low-risk patients showing a significant association between a reduced band of KM and the severity score in the adjusted sensitivity analysis (CI 95% (0.8–4.2), *p* = 0.04) [35]. Denoting this correlation of a significant relationship between insufficient KM and the severity score in univariate analysis and in sensitivity analysis only with the absence of periodontitis, it may be concluded that the negative effect of a reduced band of KM is overshadowed by the presence of periodontitis. It suggests the idea that even a sufficient width of KM cannot reduce the severity of peri-implant mucositis significantly in the presence of periodontitis.

Patients with no residual teeth display a special group among the patient sample as they show no presence of periodontitis due to the absence of residual teeth. At this point, it is important to pay attention to the history of periodontitis and thus the reason for tooth loss in the past. Continuing this idea and analyzing only the patients that did not show periodontitis in the presence and past, the significant association between the severity score and KM < 2 mm was also confirmed on patient level (CI 95% (2.1–13.6), *p* = 0.014). In contrast to the reduced width of KM gaining importance with the analysis of the subgroups, the plaque scores (mPI and API) lose relevance with the exclusion of periodontitis. This correlation supports the idea of the strength of the effect of periodontitis and the associated microbial plaque placing other confounding variables like insufficient KM or loading time in the background.

Different from our study population, Monje et al. analyzed erratic maintenance compliers claiming that adequate plaque control is the reason for the results of studies in the past, stating that there is no significant association between a reduced band of KM and peri-implant inflammation. Monje et al. showed that in implants with KM < 2 mm, clinical and radiographic parameters like PD, mBI, PI, or marginal bone loss are significantly increased [29]. According to the etiology of peri-implant diseases, the oral microbiome initiates the inflammatory response of the peri-implant tissue. It is important to keep in mind that there are no specific differences shown in the literature between the composition of mucosal and gingival biofilm neither in the condition of health nor in disease [7].

Considering the analysis of the subgroup of patients without current or former periodontitis, we could now demonstrate that insufficient KM has a significant effect on the severity of peri-implant mucositis in a patient sample not being at risk for peri-implant mucositis due to the absence of peri-implant threatening microbiome. However, these findings must be confirmed in future research with plaque sampling and microbiome analysis.

The generalizability of the study is high due to the inclusion of every implant patient during the study recruitment period. No patients were excluded. However, non-clinical patients were not included in the recruitment process. Beyond, this study shows a high degree of standardization since all implants have been examined by one examiner. Moreover, the prior sample size calculation stresses the accuracy of the study. However, due to the analysis of the subgroups, the sample size shrank in the subgroups. The significant results between a reduced width of KM and the severity of peri-implant mucositis were found in the subgroups of 59 and 21 participants representing a small proportion of the initial sample size. The number of patients in the subgroups is a considerable limitation of the present study. Furthermore, the cutoff value < 2 mm graduating dental implants with the presence of KM < 2 mm and implants with no presence of KM into the same group should cautiously be considered. We believe that a thin zone of KM (< 2 mm but > 0 mm) may have a more positive influence on peri-implant health compared with implants only surrounded by lining mucosa. A division of the study population into three groups showing either no presence of KM, or a narrow zone of KM > 0 and < 2 mm, or a wide zone of KM ≥ 2 mm is worth considering in future research. However, as most studies on the effect of keratinized mucosa on peri-implant health used a cutoff value at 2 mm, we decided to go in line with these studies to make our study outcomes comparable with the current literature. Moreover, the cross-sectional data of the present study does not give information about the temporal alteration of the condition of the implant surrounding tissue limiting the significance of our results.

Considering the recent results of the authors Oh et al., the augmentation of KM with free gingival grafts in patients with a width of KM < 2 mm displays a possibility of controlling KM as a risk indicator by reducing mucosal recession and crestal bone loss. [46].

In future, multi-center research with higher patient samples may be initiated. The predictive value of the mucositis severity score and the width of KM may be further elucidated with longitudinal data of the current patient sample.

## Conclusion

A band of KM < 2 mm is not significantly associated with the severity of peri-implant mucositis in this patient sample of university clinic outpatients. However, in subgroup analysis considering only periodontally healthy patients, a reduced band of KM is a risk indicator for the severity of peri-implant mucositis.
